# Supplementary material for: Human tumor suppressor protein Pdcd4 binds at the mRNA entry channel in the 40S small ribosomal subunit
Source: Nat Commun. 2024 Aug 8;15:6633. doi: 10.1038/s41467-024-50672-8 (PMC11310195; doi:10.1038/s41467-024-50672-8)
Supplement: Supplementary file 1 — Supplementary Imformation [file 41467_2024_50672_MOESM1_ESM.pdf]

Supplementary Imformation

Human tumor suppressor protein Pdcd4 binds at the mRNA entry channel in 40S small ribosomal subunit

Jailson Brito Querido[1,2,3,4,#,*], Masaaki Sokabe[5*], Irene Díaz-López[1*], Yuliya Gordiyenko[1], Philipp Zuber[1], Yifei Du[1], Lucas Albacete-Albacete[1], V. Ramakrishnan[1,#], Christopher S. Fraser[5,#]

[1]MRC Laboratory of Molecular Biology, Cambridge, UK.

[2]Department of Biological Chemistry and Center for RNA Biomedicine, University of Michigan, Ann Arbor, MI, USA.

[3]Life Sciences Institute, University of Michigan, Ann Arbor, MI, USA.

[4]Center for RNA Biomedicine, University of Michigan, Ann Arbor, MI, USA.

[5]Department of Molecular and Cellular Biology, College of Biological Sciences, University of California, Davis, CA 95616, USA.

*These authors contributed equally to this work.

[#]Corresponding authors. Email: jquerido@umich.edu (J.B.Q); ramak@mrc-lmb.cam.ac.uk (V.R.); csfraser@ucdavis.edu (C.S.F.).

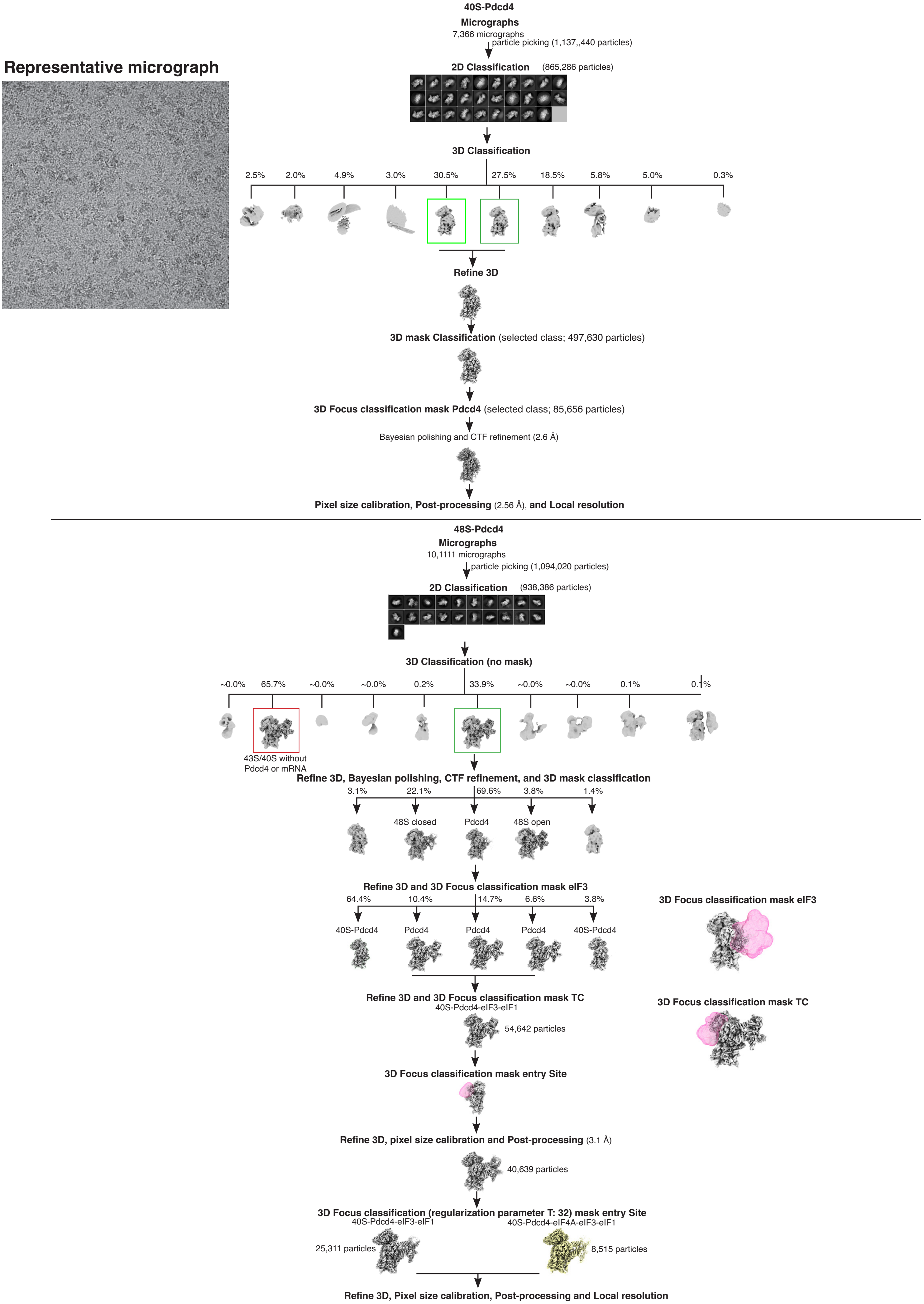

Supplementary Fig. 1: Cryo-EM data analysis of human 40S-Pdcd4 and 48S-Pdcd4. We performed independent data collection for each complex and analyzed them independently. To sort particles with Pdcd4 from particles without Pdcd4, we performed 3D focus classification. The final map of 40S-Pdcd4-eIF3-eIF1 was refined using blush regularization.

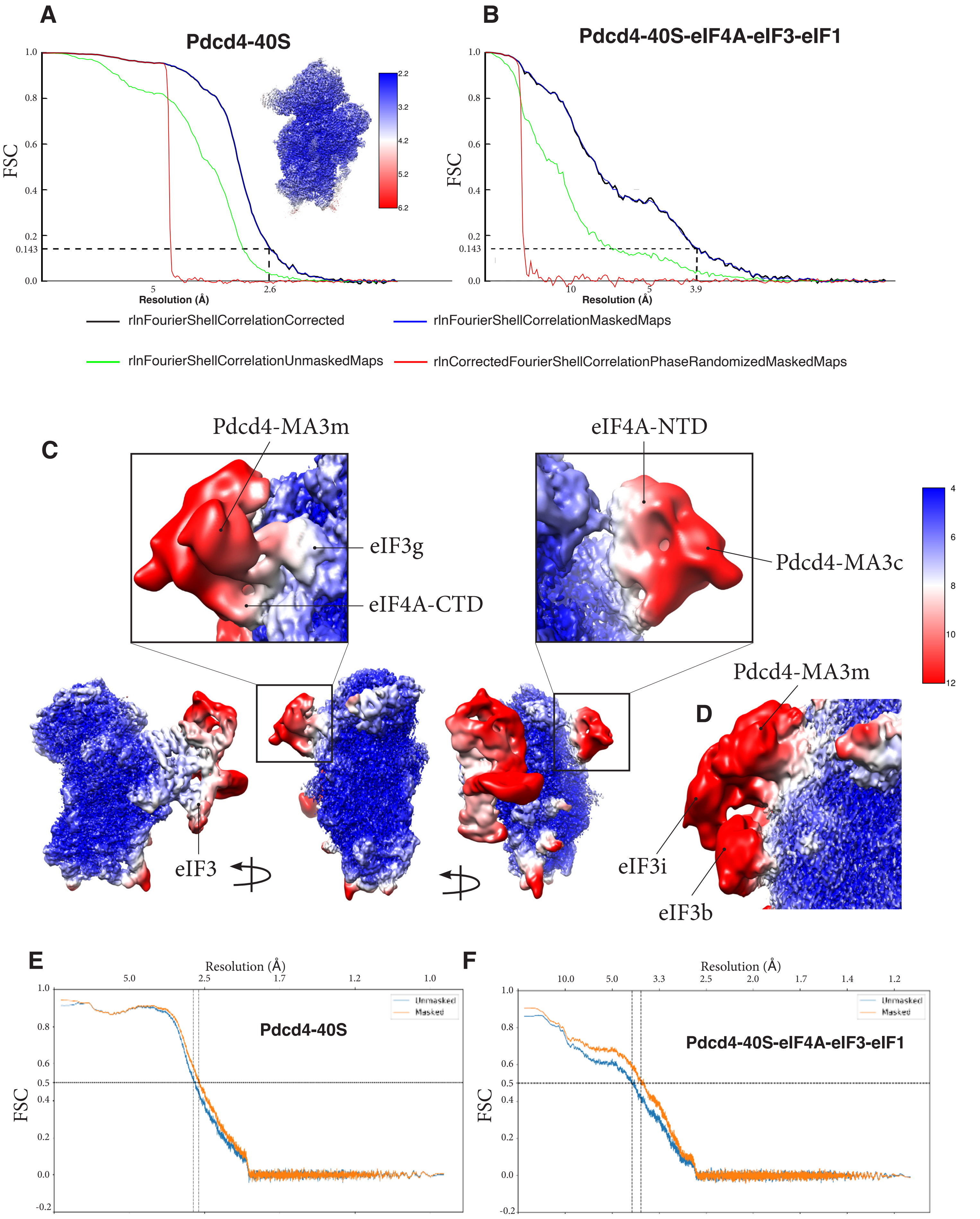

Supplementary Fig. 2: Overall and local resolution, and validation of the structure. Fourier shell correlation (FSC) curve and local resolution of the 40S-Pdcd4 (**A**). **B**) FSC curve of the 40S-Pdcd4-eIF4A-eIF3-eIF1 complex. (**C**) Local resolution of the 40S-Pdcd4-eIF4A-eIF3-eIF1 complex. The map is filtered at local resolution. D) Map at low threshold to highlight eIF3i and eIF3b (0.0035 in Chimera). **E** and **F**)The FSC curves were calculated using Mtriage as part of Phenix, comparing the atomic model with the final map, and indicating the resolution at FSC = 0.5.

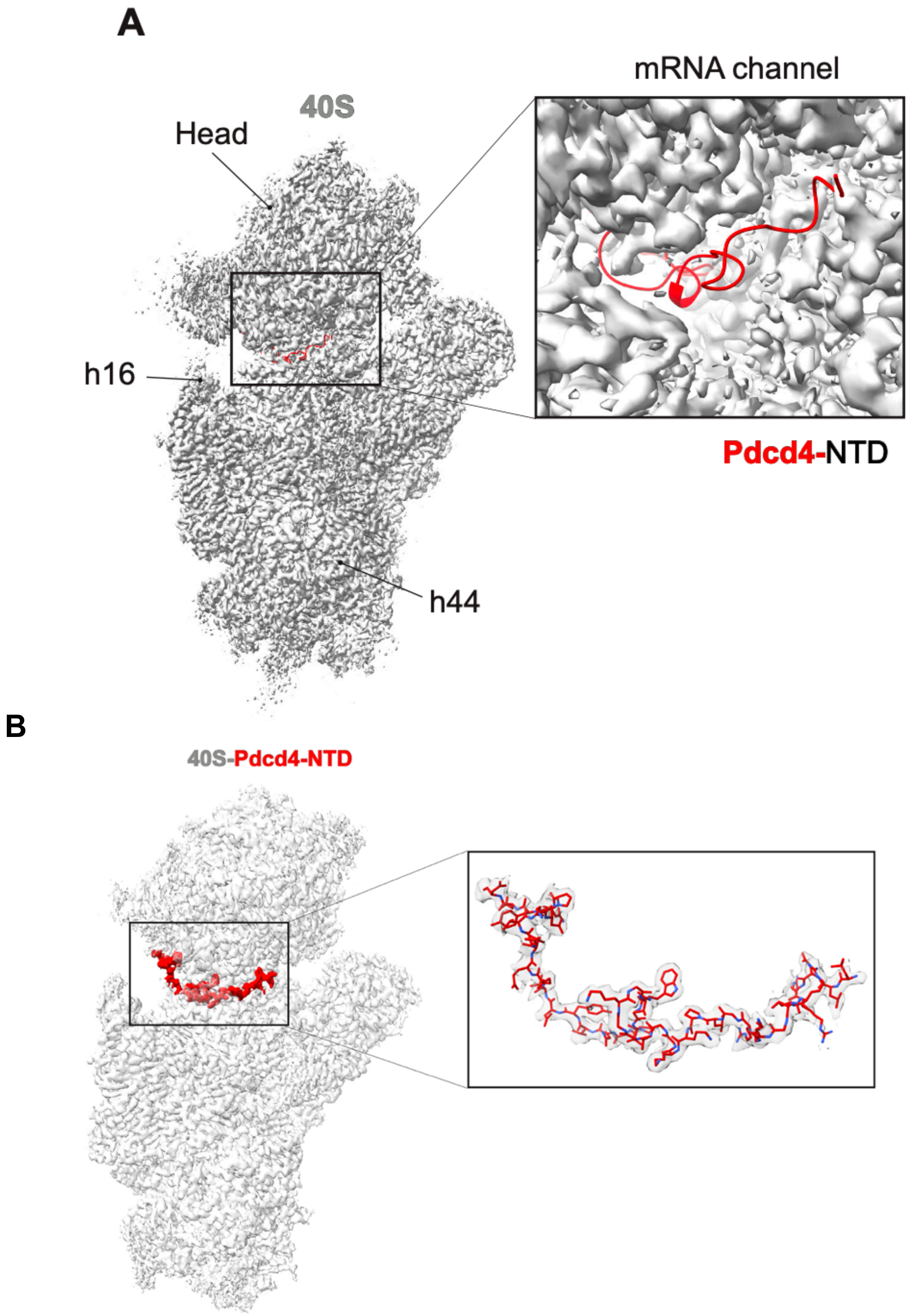

Supplementary Fig. 3: Cryo-EM reconstruction of the human 40S. (A) Cryo-EM reconstruction of human 40S collected without Pdcd4. Rigid-body fitting of the structure of human Pdcd4-40S to the density to highlight the lack of additional density in the mRNA channel. **(B)** Overview of the cryo-EM map of Pdcd4-40S showing Pdcd4-NTD (residues 99-145) in the mRNA channel.

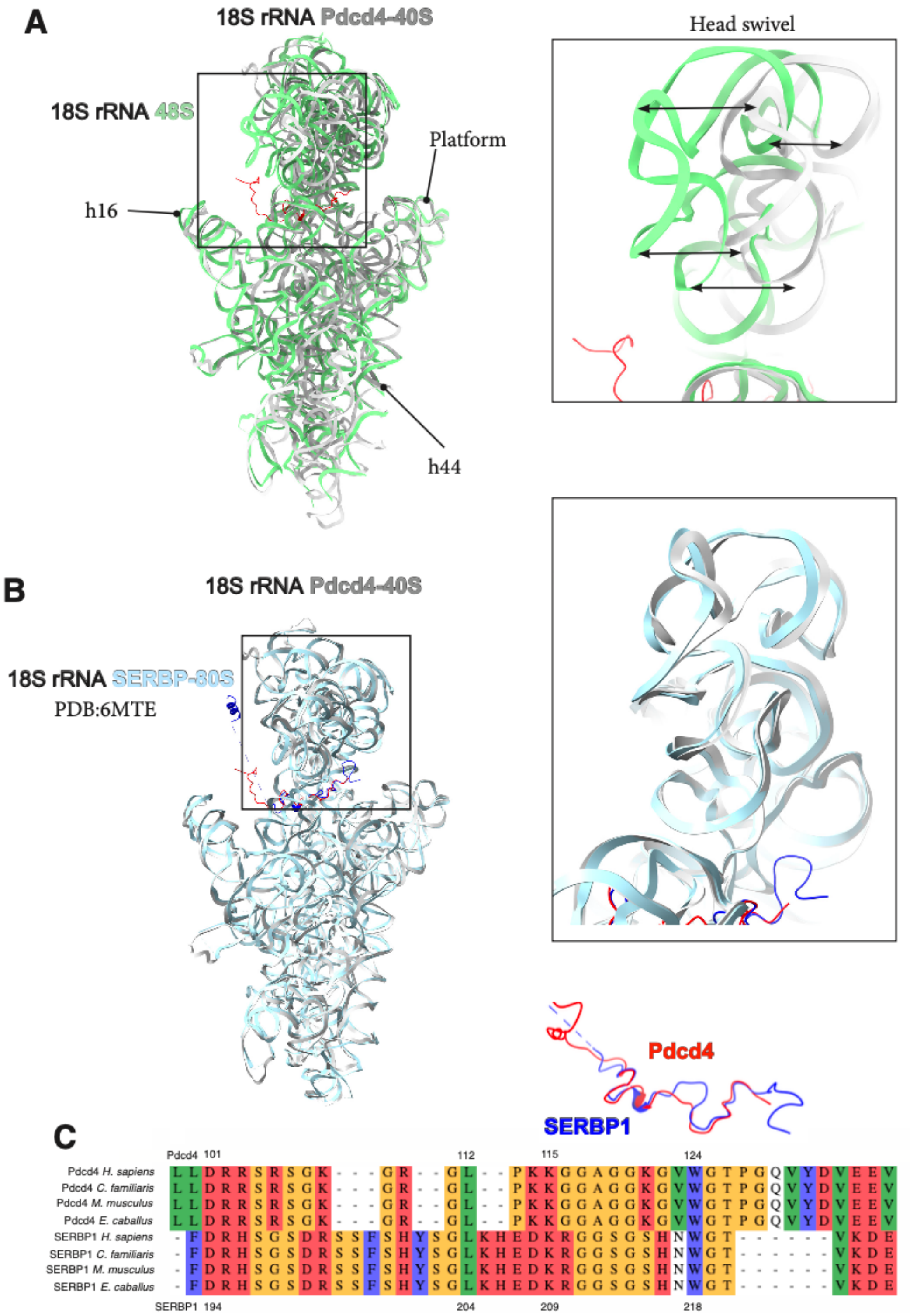

Supplementary Fig. 4: Head swivel of the 40S and structural similarities between Pdcd4-NTD and SERBP1. (A) Superposition of the structures of 18S rRNAs from human 48S [16] and Pdcd4-40S to highlight the head swivel movement observed in the structure of Pdcd4-40S. **(B)** Superposition of the structures of 18S rRNAs from Pdcd4-40S and SERBP-80S[20] complexes to highlights structural similarities. **(C)** Structure-based multiple sequence alignment of Pdcd4 and SERBP1 highlighting evolutionary conservation between the two ribosome binding domains.

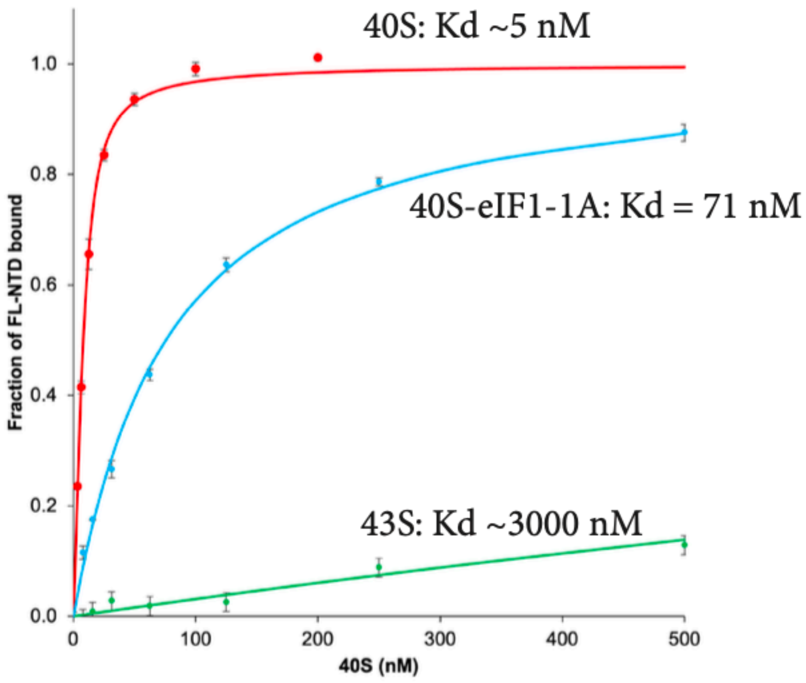

Supplementary Fig. 5: Fluorescence anisotropy binding assay with a fluorescence labelled-Pdcd4 in the presence of eIFs. The affinity of Pdcd4-NTD for the 40S subunit is reduced by eIFs and the eIF2 ternary complex. Error bars represent the mean ± SEM (n=3).

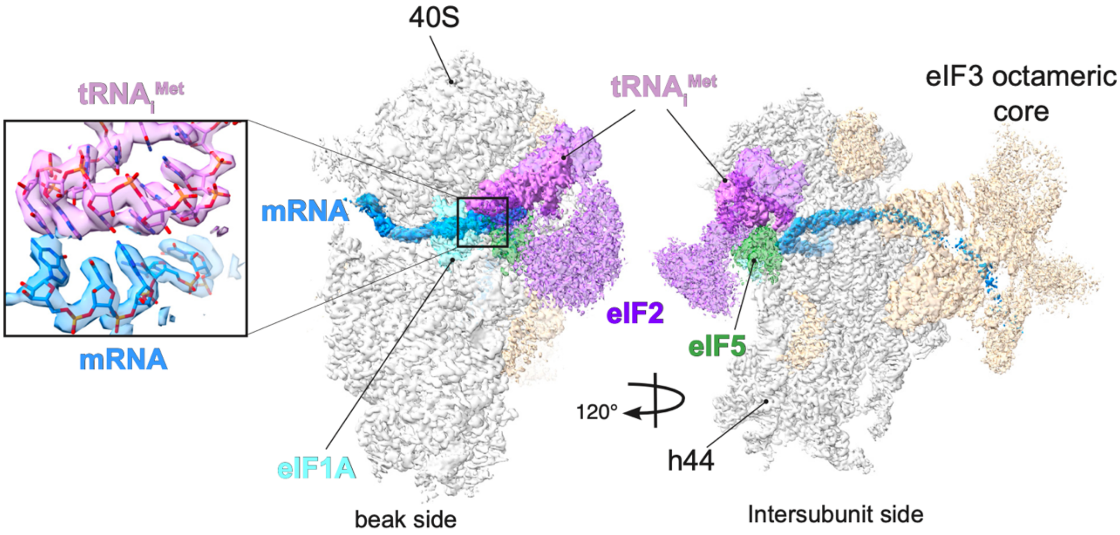

Supplementary Fig. 6: Cryo-EM 3D reconstruction of a human 48S assembled in the presence of Pdcd4. Overall view of the cryo-EM map shown in two different orientations to highlight the density of mRNA and tRNA. This 3D reconstruction lacks any density that can be attributed to Pdcd4. The sharpened maps are represented with a contour level of 0.01 using ChimeraX.

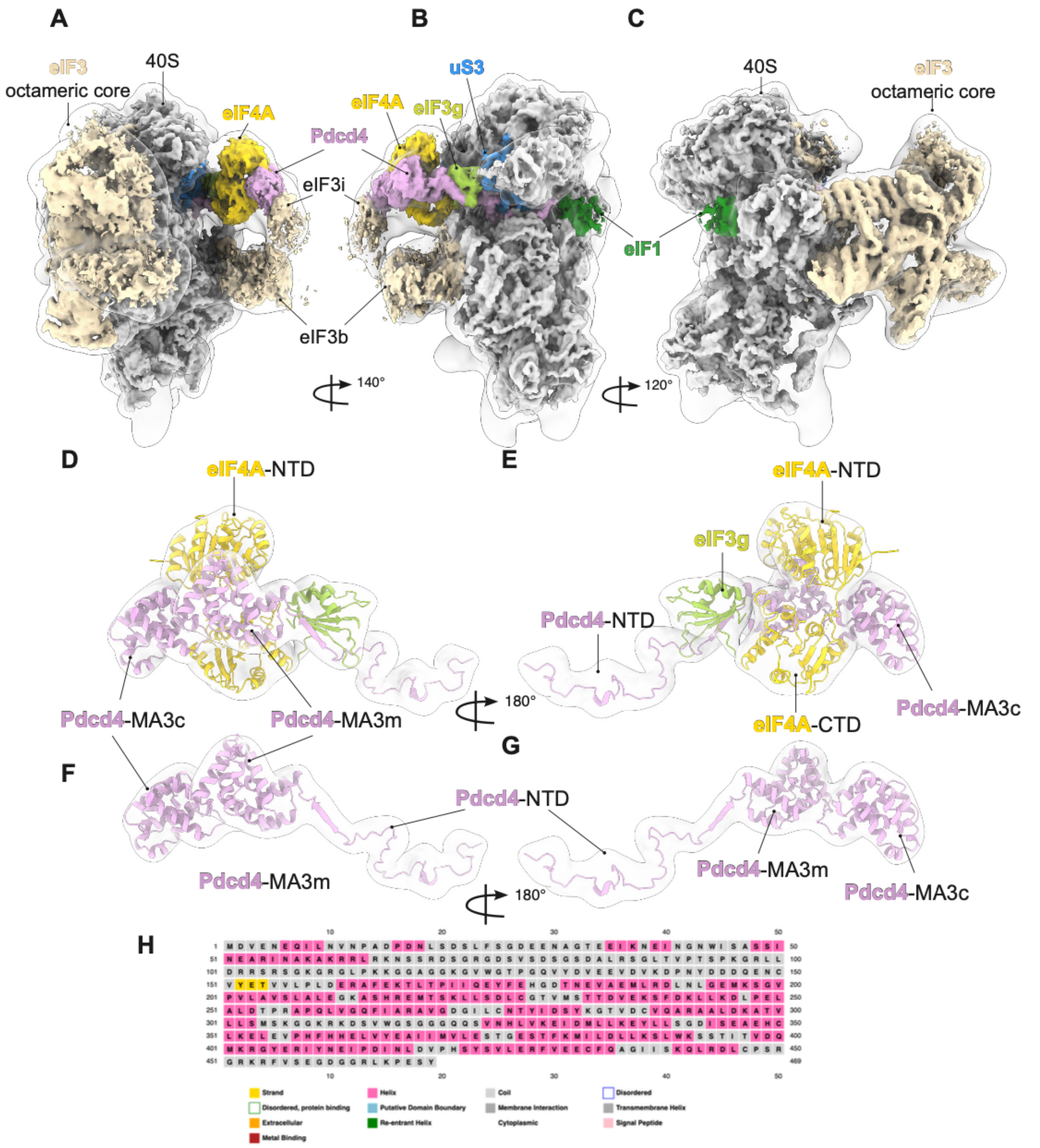

Supplementary Fig. 7: Overview of the cryo-EM density map depicted in various orientations: **(A-C)** The sharped map fitted into another map (transparent silver) filtered at a local resolution. The filtered map was determined using Relion's local resolution implementation, and smoothed by using the "surface smooth" function in ChimeraX. The sharpened maps are represented with a contour level of 0.01, except for eIF3i and eIF3b, which are represented at a contour level of 0.005 using ChimeraX. **(D-G)** The structure of Pdcd4-eIF4A (PDB:2ZU6, where one of two eIF4As is omitted)[4] was fitted onto a low-pass filtered map with a resolution of 15 Å (transparent silver). **(H)** Secondary-structure prediction map of Pdcd4 using PSIPRED, showing that residues 152-154 is predicted to form b-strand (yellow), consistent with the intermolecular b-sheet formed with eIF3g-RRM in the structure.

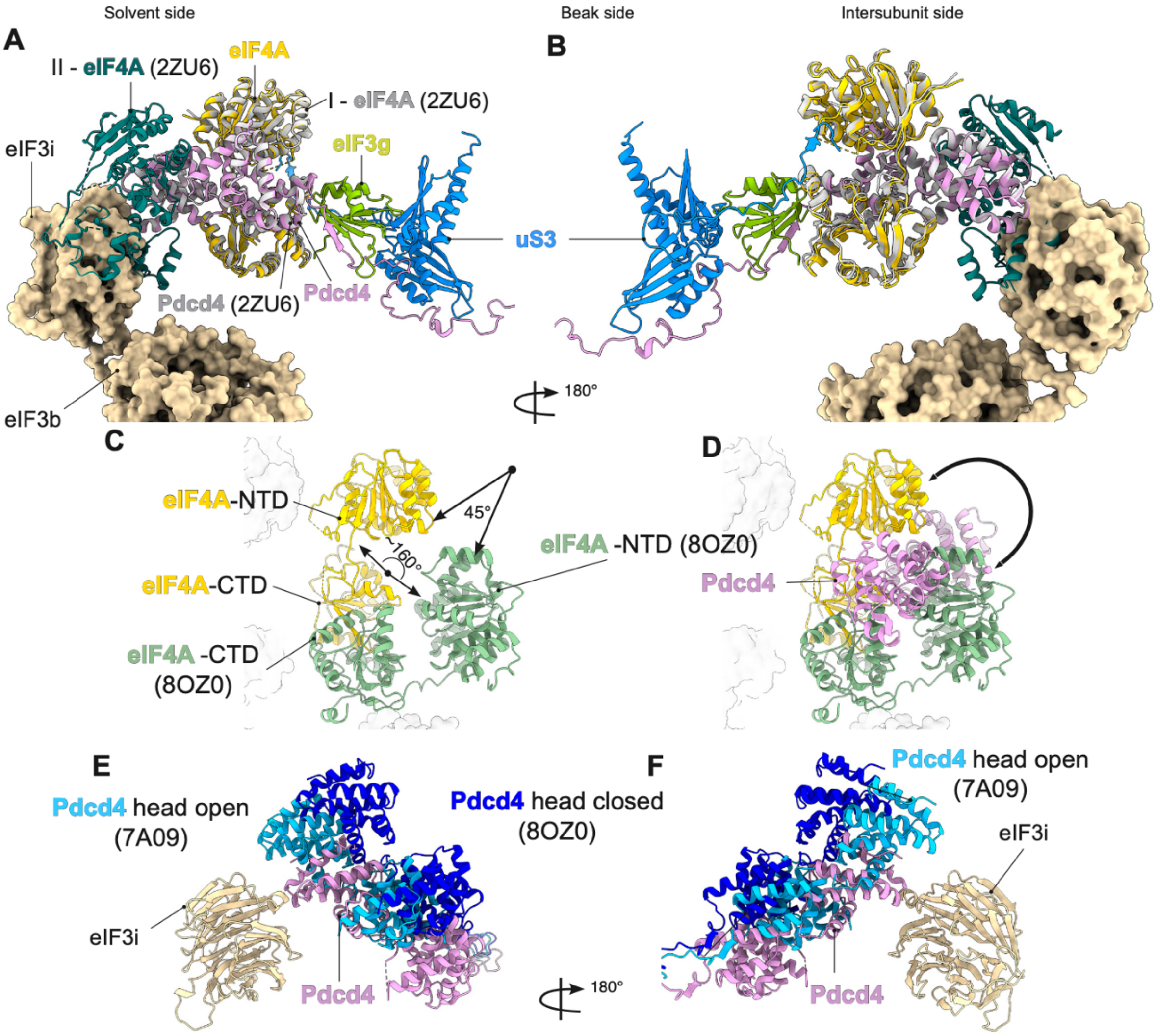

Supplementary Fig. 8: Overall structure of Pdcd4-eIF4A and conformation of eIF4A. (A-B). Superimposition of the of Pdcd4-40S-eIF4A-eIF3-eIF1 with the crystal structure of Pdcd4-eIF4A (2ZU6). Two copies of eIF4As in the crystal structure are coloured separately. The second eIF4A binding to MA3c (deep green) would clash with eIF3i. (**C-D)** Superposition of the entry site eIF4A in the 48S (PDB:8OZ0)[16] to the current structure, showing a rotation of eIF4A upon Pdcd4 binding. (**E-F)** Pdcd4-CTD superimposed with the structures of the head of 43S in open state (light blue) and the 48S in closed state (blue). In contrast to the current structure of Pdcd4-CTD (plum), those modeled on the open and closed head are further away from eIF3i (wheat). eIF4A and other molecules in the complex are omitted for visibility.

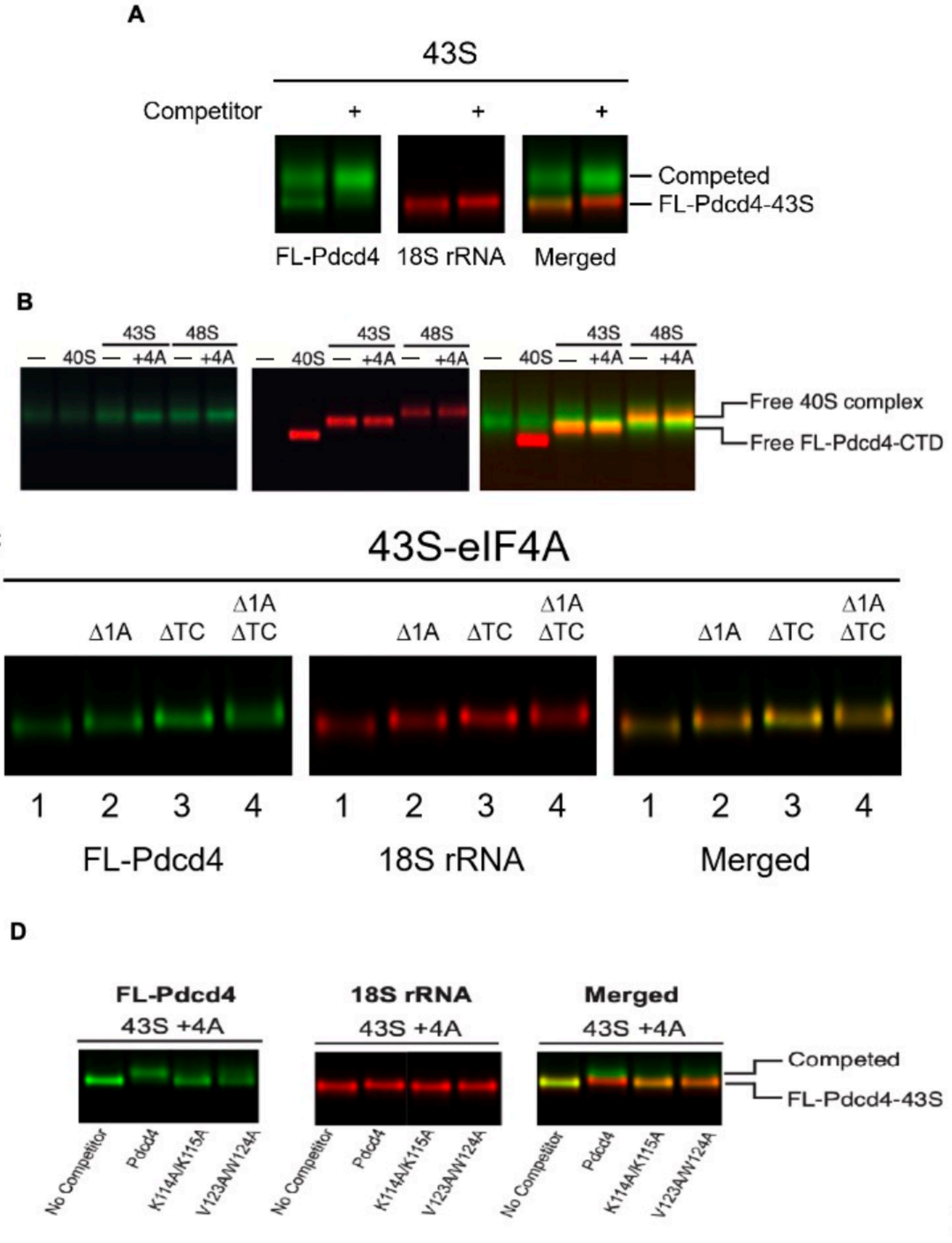

Supplementary Fig. 9: Native gel analysis of Pdcd4 binding to the 43S in the presence or absence eIF4A. Native gels are done in a similar condition as in Fig. 5**A-C**, unless otherwise indicated. (**A**) An evidence that a weak FL-Pdcd4 band overlapping with the 43S in Fig. 5A, lane 3 is indeed due to the interaction with 43S. The band is competed off and shifted back to a free form upon an addition of 30-fold excess unlabeled Pdcd4 competitor. (**B**) Pdcd4-CTD coincidentally migrates to the same position as the 43S. (**C**) Evidence that the ribosomal complex in Fig. 5A, lane 4 represents the bona fide 43S-eIF4A containing eIF1A and eIF2-TC. The partial complexes in lanes 2-4 migrate more slowly, presumably due to a more flexible conformation. (D) Comigration of Pdcd4-FL with 43S-eIF4A was analyzed in the absence (lane1) or presence of 30-fold excess of unlabeled Pdcd4 competitor (wildtype, or mutants as indicated, lanes 2-4).

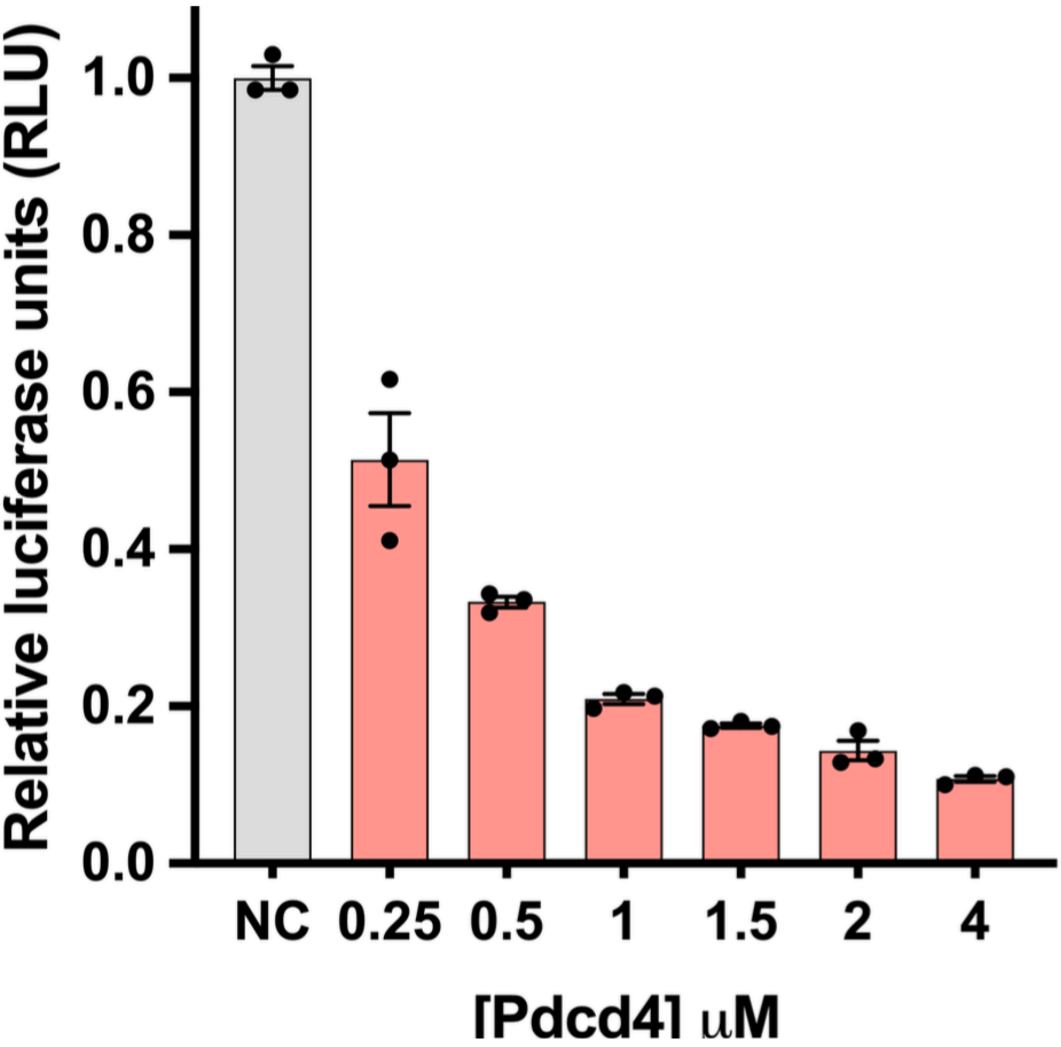

Supplementary Fig. 10: In vitro translation assay in HEK293T cells lysate. Relative luminescence activity was quantified in the presence of various concentrations of Pdcd4 (ranging from 250 to 4000 nM) and normalized to a control experiment conducted in the absence of Pdcd4 (NC). Error bars represent the mean ± SEM (n=3).

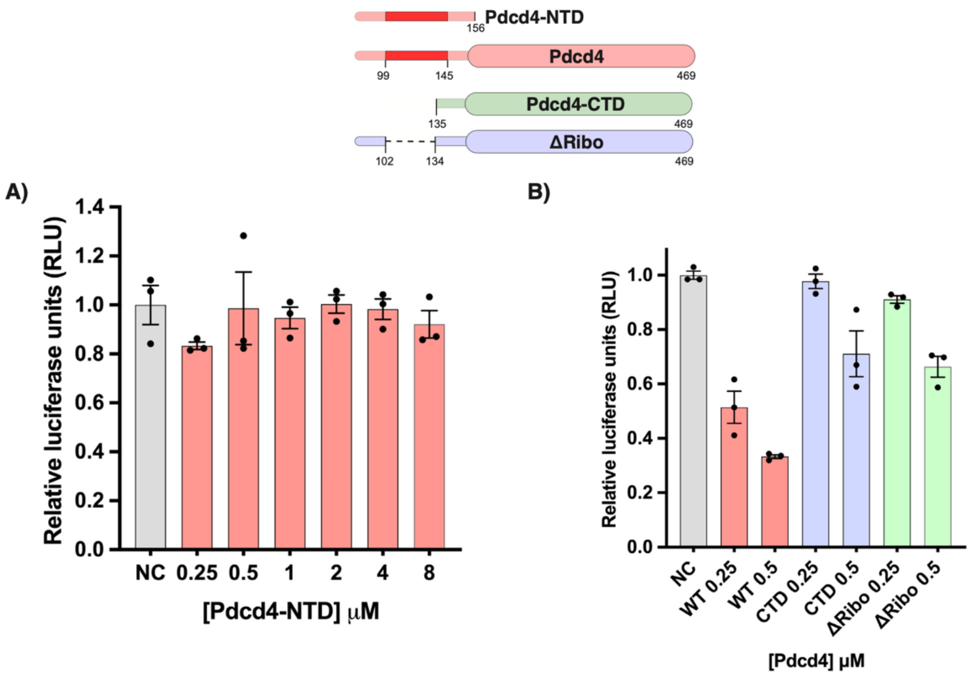

Supplementary Fig. 11: ***In vitro*** **translation assay in HEK293T cells lysate.** (**A**) Relative luciferase activity was assessed in different concentrations of Pdcd4-NTD (residues 1-156), ranging from 250 to 8000 nM. (**B**) Relative luciferase activity was assessed at concentrations of 250 nM and 500 nM for the wild type (WT), Pdcd4-CTD (lacking the first 13 4 N-terminal residues), and DRibo (lacking residues 103 to 133). A detailed titration for th e WT is provided in Supplementary Fig. 10. Error bars represent the mean ± SEM (n=3).

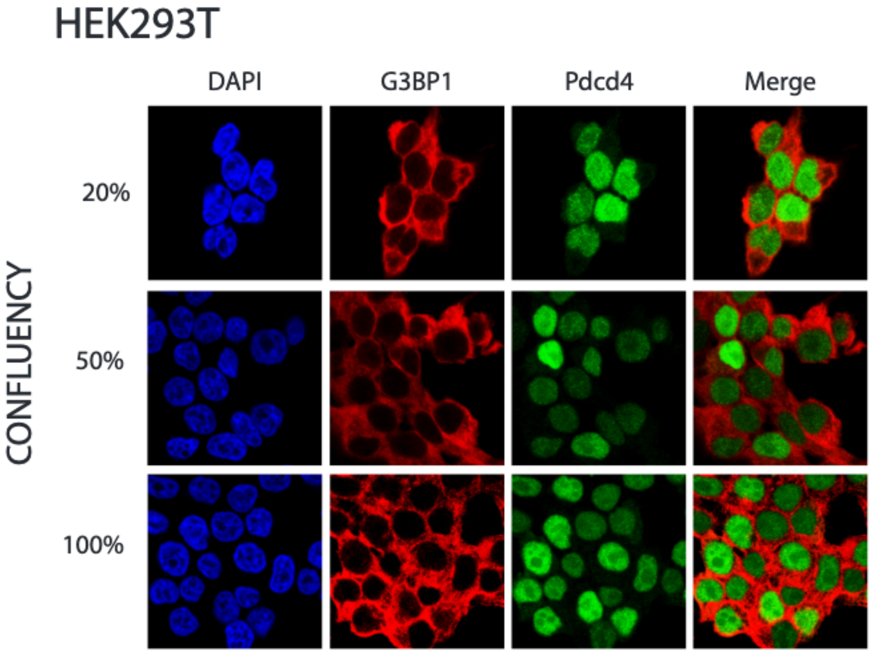

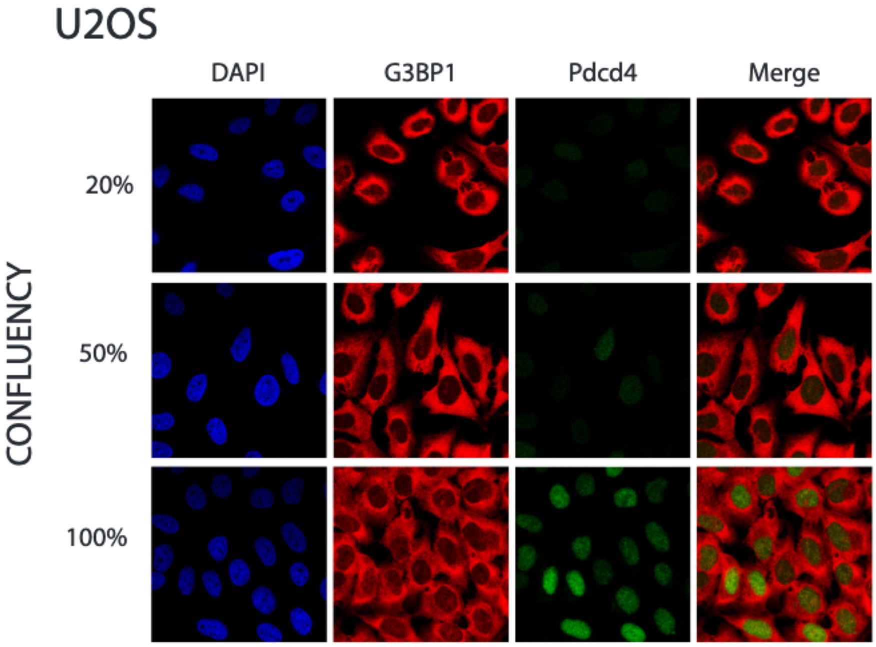

Supplementary Fig. 12: Subcellular localization of Pdcd4 in different cell lines. Immunofluorescence staining of the nucleus (DAPI, blue), cytoplasm marker (G3BP1, red), and Pdcd4 (green) in HEK293T and U2OS (tumoral cell lines) cells at increasing confluency levels.

Supplementary Table 1: Protein-protein and protein-rRNA interaction table.

Protein 1		Protein 2/rRNA	
Pdcd4	G111	R146	uS3
	G111	G145	
	K114	R143	
	G122	G112	
	V123	V115	
	W124	V115	
	W124	R116	
	G125	R116	
	Y131	Y120	
	D137	Y107	
	V138	R54	
	D140	R54	
	D140	K108	
	P141	T53	
	N142	G101	
	N142	S104	
	N142	R94	
	N142	Q101	
	D144	R94	
	D145	R54	
Pdcd4	L100	C1331	18S rRNA
	D101	C1331	
	D101	C1701	
	R102	A1489	
	R102	C1701	
	R103	C1331	
	S104	A1489	
	R105	C1331	
	S106	C1331	
	K108	G626	
	R110	C1698	
	K115	U630	
	K115	G625	
	K115	A629	
	G120	U630	

Source Data file

Uncropped scans of gels

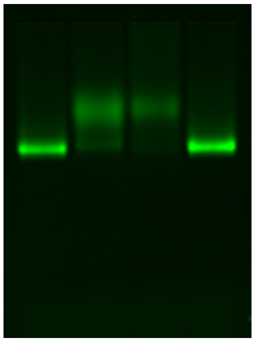

Figure 1

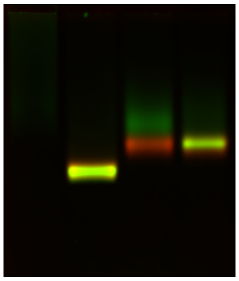

Figure 5A

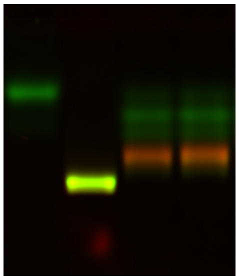

Figure 5B

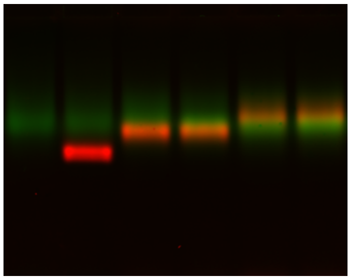

Figure 5C and S9B

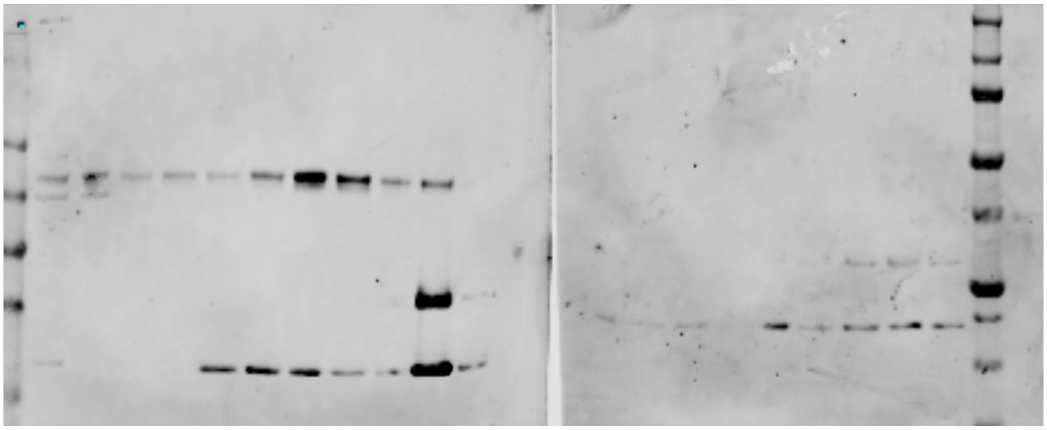

Figure 6B: Control

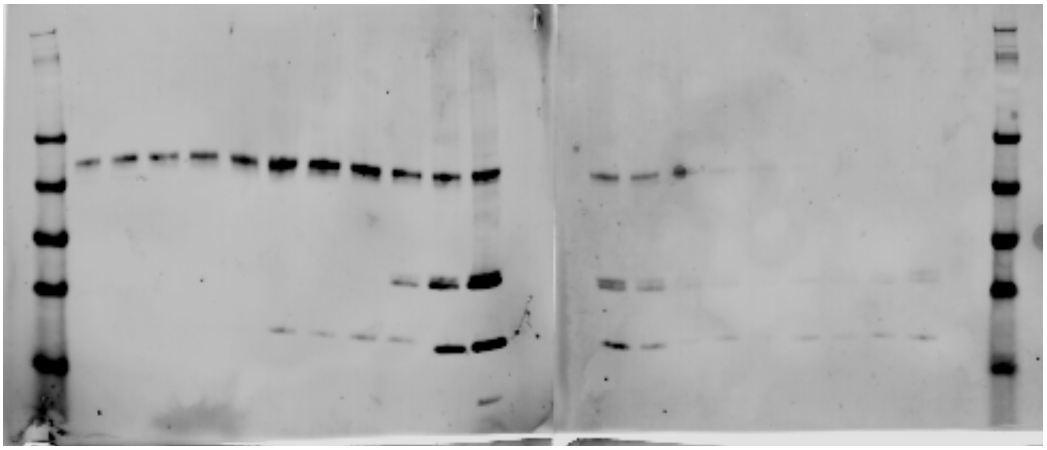

Figure 6B: Serum deprivation

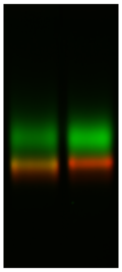

Figure S9C

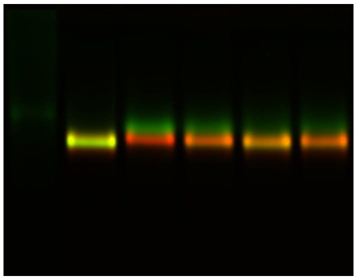

Figure S9D
